# Research on Parameter Optimization of Micro-Milling Al7075 Based on Edge-Size-Effect

**DOI:** 10.3390/mi11020197

**Published:** 2020-02-14

**Authors:** Yinghua Chen, Tao Wang, Guoqing Zhang

**Affiliations:** College of Mechatronics and Control Engineering, Shenzhen University, Nan-hai Ave 3688, Shenzhen 518060, Guangdong, China; chenyinghua2017@email.szu.edu.cn (Y.C.); tao.bit2010@hotmail.com (T.W.)

**Keywords:** micro-milling, edge-size-effect, burr, surface quality, cutting parameter optimization

## Abstract

In the process of micro-milling, the appearance of the edge-size-effect of micro-milling tools cannot be ignored when the cutting parameters are smaller than the cutting edge arc radius (*r*_0_) of the micro-milling tool or close to it, and it could easily lead to low cutting efficiency and poor surface quality of the micro-slot. Through micro-milling experiments on Al7075-T6 materials, the change of milling force in the plough zone and shear zone during micro-milling was studied, and the minimum cutting thickness (*h*_min_) range was determined to be 0.2*r*_0_–0.4*r*_0_ based on *r*_0_ of the micro-milling tool. Subsequently, the effect of *f*_z_/*r*_0_ (*f*_z_ denotes feed rate per tooth) on the top burr formation of the micro-slot, the surface roughness (*R*_a_) of the micro-slot bottom, and the milling force was studied, and a size-effect band of micro milling was established to determine the strong size-effect zone, transition size-effect zone, and the weak size-effect zone. Finally, two different *f*_z_/*r*_0_ in the strong size-effect zone and the weak size-effect zone are compared, which proves that the main purpose of the cutting parameters optimization of micro-milling is to avoid cutting parameters locating in the strong edge-size-effect zone. The above conclusions provide a theoretical basis for the selection of micro-milling cutting parameters, and an important reference in improving the surface quality of micro-milling.

## 1. Introduction

**Figure 1:**
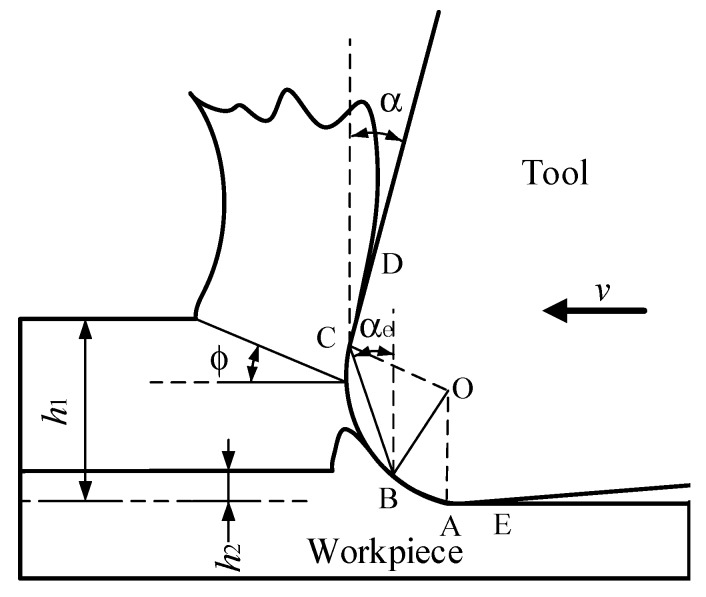
Schematic of chip formation at different cutting thickness (*h*_1_ and *h*_2_) in micro-milling.

Micro-milling technology is widely used in the manufacture of micro components and micro parts in aerospace, automobile, medical, precision mold, and other industries [1]. Different from traditional milling, the cutting edge arc radius (*r*_0_) and tool diameter (D) are in the same order of magnitude in micro-milling [2]. It is necessary to consider the influence of various size-effects caused by cutting edge arc radius, tool deflection, grain size of material, and so on [3,4,5,6]. In micro-milling, it does not follow that the smaller the cutting parameters, the better the surface quality. In the micro-milling process, the ratio *f*_z_/*r*_0_ of the feed rate per tooth (*f*_z_) to the cutting edge arc radius (*r*_0_) is relatively small, which results in the proportion of material removed by the cutting edge arc surface (AC section) instead of the front cutting surface (CD section), and the elastic deformation of material caused by the strengthening contact between the back cutting surface (AE section) and the workpiece surface, which results in more obvious scraping, squeezing, and ploughing of the cutting edge arc surface on the workpiece surface (as shown in Figure 1). This kind of size-effect is so-called the edge-size-effect in this paper, because the cutting parameters decrease to the size that is close to *r*_0_. The edge-size-effect will lead to problems of poor surface quality, increased number and size of burrs, and increased wear of the micro-milling tool [7,8,9]. When *f*_z_ in the micro-milling is larger than *r*_0_, the rake face of the tool really starts to participate in cutting, and as the proportion of rake face participating in cutting continues to increase, it gradually shows a similar cutting mechanism to macro cutting [10].

Kiswanto et al. (2014) [11] studied the size-effect in micro-milling, and found *f*_z_ and *r*_0_ had the greatest impact on the surface quality of micro machining. Kang et al. (2008) [12] considered that *r*_0_ has a great influence on the micro-milling force in all directions in the micro-milling process; the relationship between the cutting edge arc radius (*r*_0_) and the minimum cutting chip thickness (*h*_min_) decides whether chips are generated in the micro-milling process. In the process of micro-milling, when *h*_min_ is equal to *r*_0_ or close to it, the positive rake angle α of the cutting edge changes to the negative rake angle α_e_ [13], and elastic deformation occurs between the workpiece and the tool; however, no chips are formed, the tool mainly produces compression and friction force on the workpiece surface, and the ploughing phenomenon occurs. Therefore, in order to form chips continuously and stably in the cutting process, it is necessary to increase the cutting thickness (*h*_2_) (Figure 1), so that *h*_min_ may experience from elastic plastic deformation to shear deformation [14,15]. Takacs & Vero (2007) [16] found that *h*_min_ has a high correlation with *f_z_*. Other researchers [4,17,18] reported that *h*_min_ can be determined by analyzing the cutting force without considering the workpiece material, and *h*_min_ varies from 1/4 *r*_0_ to 1/3 *r*_0_.

In summary, most researchers in the research field of micro-milling firstly determine the *h*_min_ range by experiment or simulation, and then use the qualitative analysis method to study the influence of micro-milling surface quality and the optimization strategy of cutting parameters. However, there is little research on the optimization of cutting parameters based on the quantitative analysis of the size-effect on cutting edge, so as to reduce burr and surface roughness. The essence of the edge-size-effect is that the material removal mode of micro-milling gradually changes from plough and friction to shear with the increase of *f*_z_/*r*_0_. This means the elastic deformation of the material being machined can also be transformed into plastic deformation. The friction resistance between the tool and the workpiece material is reduced, and the cutting chip can be generated more easily. The amount of residual chips at the bottom and side of the micro-slot will affect the surface quality and burr formation in micro-milling.

In this paper, the range of *h*_min_ is determined by studying the milling force variation in plough and shear areas during micro-milling. Then, the influence of *f*_z_/*r*_0_ on the top burr size of micro-slot, surface roughness (*R*_a_) of the micro-slot bottom, and the micro-milling force is studied, and the strong size-effect zone, the transition size-effect zone, and the optimal size-effect zone are determined; thereby, the size-effect band of micro-milling is established. Finally, the size-effect zones of different *f*_z_/*r*_0_ values are compared to optimize the micro-milling cutting parameters, so as to avoid the cutting parameters in the strong edge-size-effect zone. This study provides the theoretical basis for the configuration of micro-milling cutting parameters and technical reference for improving the surface quality of micro-milling.

## 2. Experimental Design

### 2.1. Determination of the Minimum Cutting Thickness (h_min_)

In micro-milling, the value of *h*_min_ can be identified and estimated indirectly. Yuan et al. (1996) [19] assumed that there is a separation point between the plough cutting area and shear cutting area on the cutting edge arc, and by studying the relation between the force ratio of *F*_x_/*F*_y_ and *h*_min_, *h*_min_ of about 20%–40% of *r*_0_ was obtained. Liu et al. (2005) [20] established the minimum chip thickness model considering the thermal softening and strain strengthening effects of workpiece materials, and found that *h*_min_ of 1040 steel and Al6082-T6 aluminum were 20%–35% and 35%–40% of *r*_0_, respectively.

**Figure 2:**
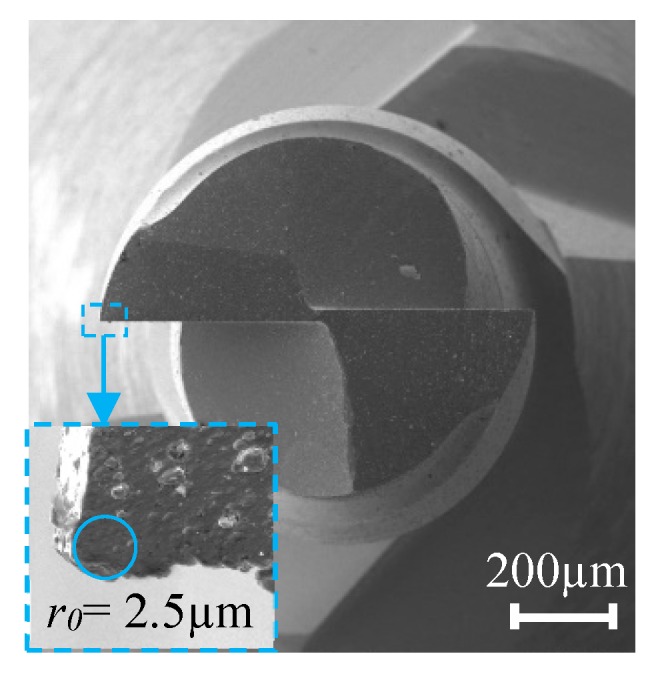
A 1 mm diameter flat end milling tool and edge morphology under SEM (FEI Quanta 450 FEG).

**Figure 3:**
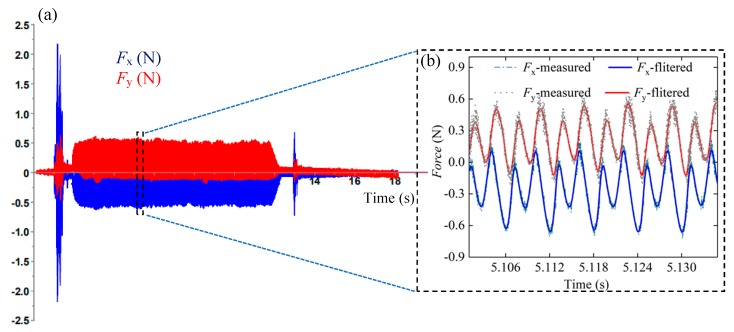
Milling forces in the feed direction (*F*_y_) and the step direction (*F*_x_): (**a**) original milling force, (**b**) comparison of measured and filtered milling force.

The morphology of the micro-milling tool used in the test is shown in Figure 2, and *r*_0_ of the micro-milling tool is about 2.5 μm. From the conclusion of other research [19,20], different workpiece materials cause different *h*_min_, and theoretically, the minimum chip thickness can be approximately 0.2 *r*_0_–0.4 *r*_0_. Figure 3 shows the measured milling force variations during the micro-milling process in the feed direction (*F*_y_) and the direction perpendicular to it (*F*_x_). Figure 3a is a set of original milling force data measured during micro-milling, and Figure 3b is a partial enlarged view of the measured milling force. Figure 3b shows that the filtered and measured milling force (*F*_x_ and *F*_y_) have similar variations and closely matched amplitude levels. The value of *F*_x_ and *F*_y_ corresponding to different *f*_z_ can be obtained by calculating the average value of the absolute amplitude of 10 peaks and 10 valleys in the 5 groups of cutting force cycles. The actual measured cutting force amplitude is small, and a low-pass filter setting of 800 Hz can actively filter out the influence of high-frequency noise signal generated by machine tool vibration and other interference factors on the measurement of the micro-milling force.

**Figure 4:**
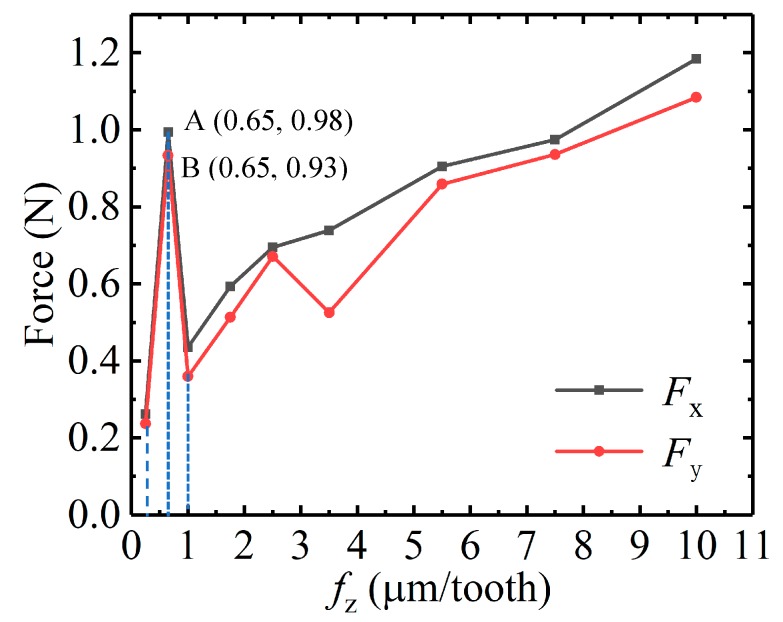
Milling force *F*_x_ and *F*_y_ with respect to *f*_z_ in micro-milling Al7075.

Figure 4 shows the curve of milling force *F*_x_ and *F*_y_ with respect to *f*_z_ during micro-milling Al7075. When *f*_z_ changes in the range of 0.2 µm/tooth–1 µm/tooth, the micro-milling force increases first and then decreases with the increase of *f*_z_, presenting a non-linear forms [21,22], which indicates the edge-size-effect appears in the micro-milling process. When *f*_z_ is 0.65 µm/z, *F*_x_ and *F*_y_ reach peaks A and B, respectively; therefore, *h*_min_ can be determined by the milling force to be about 0.65 µm (as shown in Figure 4), which is in the range of 0.2 *r*_0_–0.4 *r*_0_ of the theoretical result of the *h*_min_, thus the theoretical value is basically consistent with the experimental result. Therefore, *h*_min_ in this paper is determined to be 0.5 μm–1 μm, which provides a theoretical basis for the follow-up test to study the effects of size-effect and the selection of cutting parameters under different size-effect bands.

### 2.2. Single Factor Micro-Milling Test

**Figure 5:**
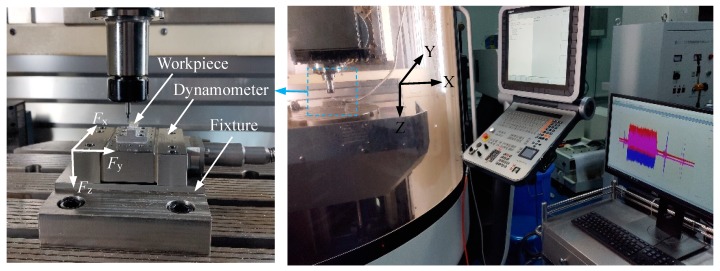
Experimental setup of micro-milling.

The single-factor test of the micro-milling slot was performed on a five-axis linked mill machining center (DMU 40 eVo, DMG MORI, Bielefeld, Germany). The power of the machine tool is 1.5kw, whose maximum spindle speed is 18000 r/min, and the positioning error of the machine tool is 3 μm.. The micro-milling experimental setup is shown in Figure 5.

Dry cutting was employed in the experiment; the workpiece material is aluminum alloy (Al7075-T6). The milling forces in the direction of *F*_x_, *F*_y_, and *F*_z_ were measured in real time through a Kistler-9119AA1 dynamometer. The sampling frequency of the dynamometer was set to 36 KHz, which can ensure capturing enough force signal data point. A two-edge TiAlN-coated flat end milling tool (MX230, NS TOOL, Tokyo, Japan) was employed in the milling experiment; the material of the micro-milling tool is superhard alloy, the diameter of which is 1 mm, while the front angle, flank angle, and helix angle are 12°, 5°, and 30°, respectively.

Under the given parameters of the micro-milling tool, workpiece material, machining machine, and cutting conditions, the change of the cutting parameters usually has an important influence on the burr size and surface roughness (*R*_a_) of the micro-slot surface. Considering the influence of the edge-size-effect and *h*_min_ on the micro-milling process, the cutting parameter settings in the experiment are listed in Table 1. The experimental groups N1–N8 are used to study the influence of the edge-size-effect on the micro-milling, while experimental groups N9–N24 are mainly used to study the influence of cutting parameters on the size of the top burr and *R*_a_ of the micro-slot surface under the strong and weak size-effect zone.

**Figure 6:**
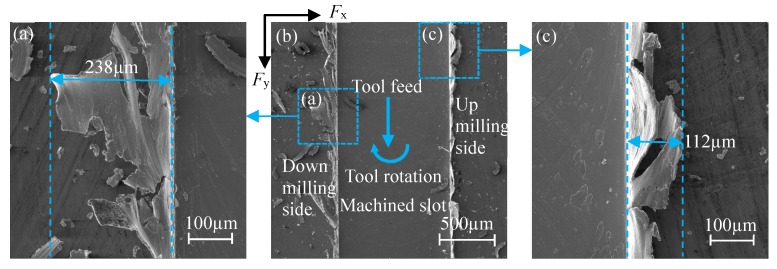
Measurement of micro-slot burr morphology and its top burr width: (**a**) down milling side, (**b**) micro-slot figure, and (**c**) up milling side.

Finish milling was conducted on the workpiece surface to be machined before the test, which can eliminate the effects of the preparation process of the workpiece material and the surface defects on the surface quality and the actual cutting depth. Under the 100× and 450× lenses of SEM (Quanta 450 FEG, Field Electron and Ion Company, Hillsboro, OR, USA), the shape of micro-slot burr and the width of top burr can be analyzed, as shown in Figure 6, where (a) is the top burr on the down milling side, (b) is the micro-slot after machining, and (c) is the top burr on the up milling side. *R*_a_ of the micro-slot bottom can be measured by Keyence’s VK-X series laser scanning confocal microscope.

## 3. Results and Discussions

### 3.1. Classifications of Size-Effect Band in Micro-Milling

The size-effect band of micro-milling refers to the workpiece material Al7075 that is affected by the edge-size-effect of the micro tool in the micro-milling process, thereby the shape and size of micro-slot burr and the surface roughness of slot bottom change with *f*_z_/*r*_0_.

**Figure 7:**
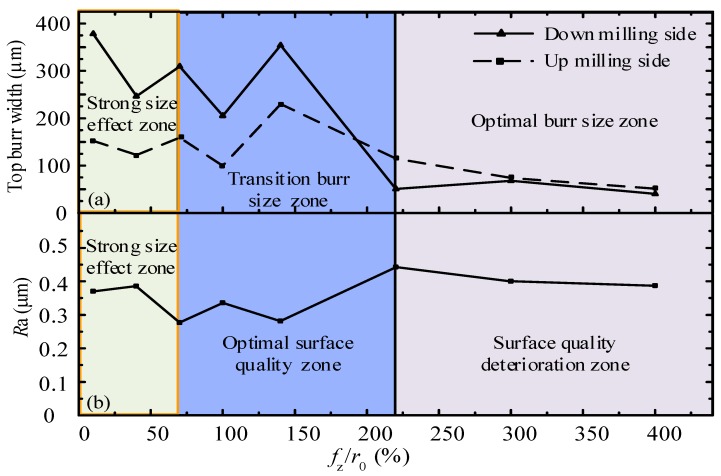
Variations of the top burrs’ width and *R*_a_ of the micro-slot bottom in different size-effect band. (**a**) top burr width with respect to *f*_z_/*r*_0_, (**b**) surface roughness width with respect to *f*_z_/*r*_0_.

As observed from Figure 7, when *f*_z_/*r*_0_ in the strong edge-size-effect zone is less than 70%, the width of the top burr is large, and the surface roughness of the micro-slot bottom is large. This is because, when *f*_z_ is close to *r*_0_, the proportion of the cutting material in front of the cutting tool is small, and the size-effect of the cutting edge is significant. When *f*_z_/*r*_0_ in the transition size-effect zone is in the range of 70%–220%, the edge-size-effect has a significant effect on the top burr, and the width of the top burr has a great change, however, the surface quality of the micro-slot bottom is good, and *R*_a_ value is relatively small. Therefore, an optimal surface quality zone is defined in this transition size-effect zone. When *f*_z_/*r*_0_ in the weak edge-size-effect zone is greater than 220%, *f*_z_ is far greater than *r*_0_. The micro-milling is less affected by the edge-size-effect, similar to the traditional milling process, and the *R*_a_ value of the slot bottom is larger. However, the burr is mainly in contact with the front face of the cutting tool, while the workpiece material is mainly plastic deformation, the burr is more easily pushed off, and the width of the burr at the top of the micro-slot becomes smaller. Therefore, the weak edge-size-effect zone can be defined as the optimal burr size zone. The large top burr width mainly occurs in the strong edge-size-effect zone and transition burr size zone (Figure 7a), and the large *R*_a_ of the micro-slot bottom mainly occurs in both the strong edge-size-effect zone and the surface quality deterioration zone (Figure 7b).

### 3.2. Optimization of Micro-Milling Parameters Based on Top Burr Morphology

**Figure 8:**
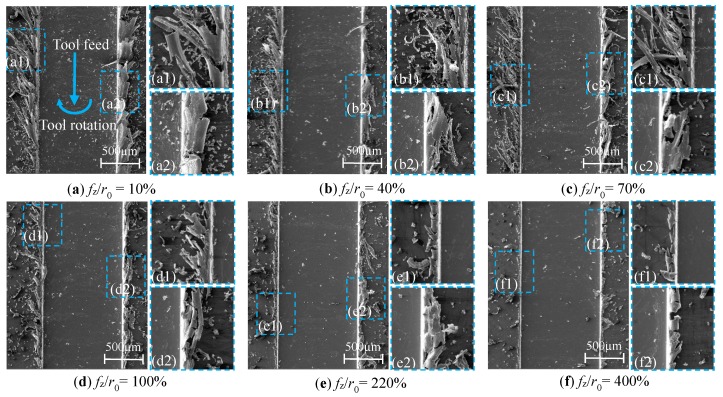
Variations of micro-slot and burr morphology in the size-effect zone. (**a**) *f*_z_/*r*_0_ = 10%; (**b**) *f*_z_/*r*_0_ = 40%; (**c**) *f*_z_/*r*_0_ = 70%; (**d**) *f*_z_/*r*_0_ = 100%; (**e**) *f*_z_/*r*_0_ = 220%; (**f**) *f*_z_/*r*_0_ = 400%.

By observing the top burr morphology of the micro-slot, the influence of *f*_z_ on the burr morphology is studied; the micro-milling test is designed as N1–N8 in Table 1. The micro-slot and burr morphology images captured by SEM are shown in Figure 8.

When *f*_z_/*r*_0_ is less than 70%, the micro-milling process is in a strong size-effect zone. Along with the decrease of *f*_z_/*r*_0_, the top burrs’ width of both the down milling side and up milling side decreases first and then increases, as shown in Figure 7a. The top burrs of the down milling side present a whisker-shaped morphology (Figure 8a1–c1), while the top burrs of the up milling side present a block-shaped morphology (Figure 8a2–c2). These phenomena show that the micro-milling cannot generate chips because of the influence of the strong edge-size-effect. However, the materials in contact with the tool are squeezed and scratched continuously along the micro-slot edge to form strip burr owing to the good plasticity and ductility of the aluminum alloy material.

When *f*_z_/*r*_0_ is larger than 70% and less than 220%, micro-milling is in the transition size-effect zone, and the top burr width in the down milling side and up milling side of the micro-slot is larger in size and deviations, as shown in Figure 7a. This is because of the reduction of elastic recovery for the machined surface, the ploughing effect area reduces while the shear effect area increases, chips cannot be stably generated, chips reaching the edge of the micro-slot cannot be separated timely, and then the top burrs are formed. From Figure 8a–d, it is found that the top burr width in the down milling side is significantly larger than the top burr width in the up milling side, and the top burr width fluctuates with the decrease of *f*_z_/*r*_0_. When *f*_z_/*r*_0_ is 140%, Figure 7a reports that the maximum value of the top burr width in the down milling side and the up milling side is 388 μm and 265 μm, respectively, which indicates that the shape and size of the formed chips are not consistent, thereby the formation of burrs owns uncertainty, and the top burr width has great changes. The top burrs in the down milling side are mainly in a tearing flag shape (Figure 8d1), while the top burrs in the up milling side are in a continuous serration shape (Figure 8d2).

When the *f*_z_/*r*_0_ value is greater than 220%, Figure 7a shows that micro-milling is in the optimal burr size selection zone, where the edge-size-effect has the least effects. The top burrs on both sides of the down and up milling are small and few, and the top burrs’ width decreases with the increase of *f*_z_/*r*_0_, as shown in Figure 8e–f. Further, the top burrs of the down milling side and up milling side mainly present a curly and wavy shape, as shown in Figure 8f1–f2. When *f*_z_/*r*_0_ is 220%, Figure 8e1–e2 show that the top burr width in the down milling side begins to be smaller than the top burr width in up milling side, which indicates that the top burr in the down milling side breaks away from the influence of the edge-size-effect zone before the top burr in the up milling side.

*(1) Influence of Axial Cutting Depth on the Burr Size-Effect Band*

The cutting parameters for the micro-milling straight slot test are shown in the N9–N16 groups in Table 1. Under the two conditions of *f*_z_/*r*_0_ = 40% (strong size-effect zone) and *f*_z_/*r*_0_ = 250% (weak size-effect zone), observing the top burr morphology of the micro-slot when the axial cutting depth (*a*_p_) was changes, the influence of *a*_p_ on the burr size-effect band is studied.

**Figure 9:**
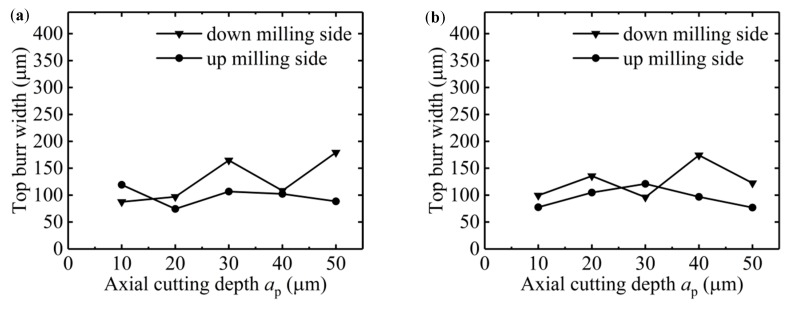
Change of the top burr width of micro-slot with *a*_p_ (N9–N16): (**a**) *f*_z_/*r*_0_ = 40%, (**b**) *f*_z_*/r*_0_ = 250%.

The change of *a*_p_ in different size-effect zones has different effects on the burr morphology in the two sides of the micro-slot. As shown in Figure 9a,b, the top burr width in the down milling side changed from 90 µm to 179 µm, while the top burr width in the up milling side remained around 100 µm, which shows that the change of *a*_p_ has a more significant effect on the top burr size of the down milling side. This is because the tool cutting depth in the down milling side changes from the maximum to the minimum, and the direction of the chip flow-out is the same as the moving direction of the workpiece, thus it is easier to slit the top burr and generate long chips.

**Figure 10:**
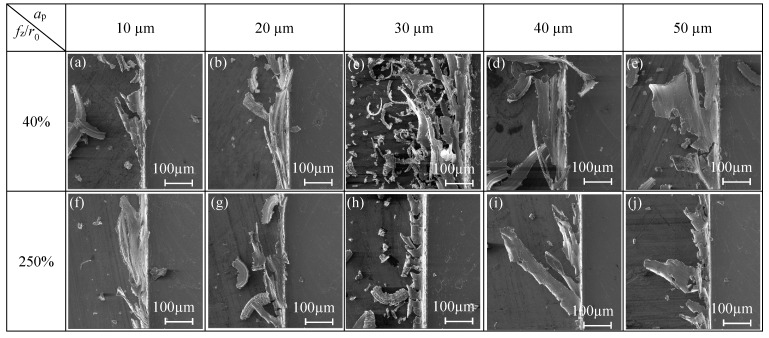
Changes of top burr morphology in the down milling side with respect to *a*_p_ in the size-effect zone (N9–N16).

**Figure 11:**
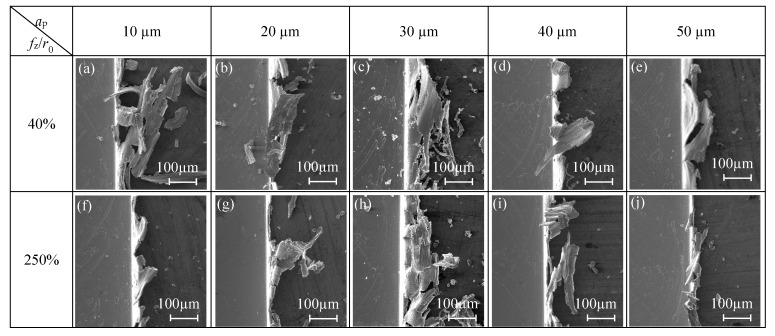
Changes of top burr morphology in the up milling side with respect to *a*_p_ in the size-effect zone (N9–N16).

*f*_z_/*r*_0_ = 40%, which means the micro-milling is in the strong size-effect zone (Figure 7), when the cutting depth is less than 20 μm, the top burr width in down milling side is close to 100 μm. When the axial cutting depth continues to increase to 50 μm, the top burr width in the down milling side increases to 179 μm and with obvious fluctuation, while the top burr width in the up milling side of the micro-slot decreases slightly, as shown in Figure 9a. The top burrs in both the down milling side and up milling side mainly present lager patches or a striped shape, as shown in Figure 10a–e and Figure 11a–e, respectively.

*f*_z_/*r*_0_ = 250%, which means the micro-milling is in the weak edge-size-effect zone, and the change of axial cutting depth has little effect on the top burr size. The change range of the top burr width of the micro-slot is small and does not exceed 30 μm in both the down milling side or up milling side, and the top burr in the up milling side increases first and then decreases with the increase of the axial cutting depth (Figure 9b). Therefore, the separation of micro-milling from the strong size-effect zone could be realized by increasing the axial cutting depth to reduce the top burr or selecting a smaller *f*_z_/*r*_0_ value, in order to reduce the optional range of cutting parameters. The top burr morphology in the down milling side and up milling side mainly presents as discontinuous serration or wavy (Figure 10f–j and Figure 11f–j, respectively).

*(2) Influence of Cutting Speed on Burr Size-Effect Band*

The cutting parameters for the micro-milling straight slot test are shown in the N17–N24 group in Table 1. Under the two conditions of *f*_z_/*r*_0_ = 40% (strong edge-size-effect zone) and *f*_z_/*r*_0_ = 250% (weak edge-size-effect zone), observing the top burr morphology of the micro-slot when cutting speed(*v*) changes, the influence of *v* on the burr size-effect band is studied.

**Figure 12:**
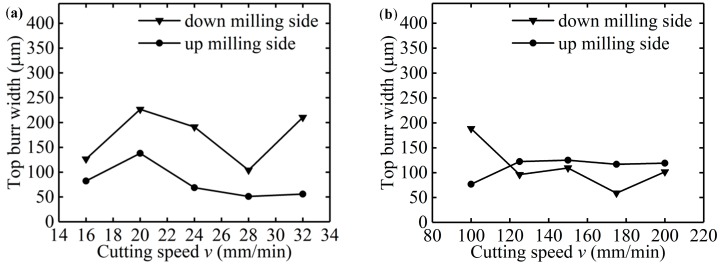
Changes of top burr width of the micro-slot with *v* (N17–N24): (**a**) *f*_z_/*r*_0_ = 40%; (**b**) *f*_z_*/r*_0_ = 250%.

**Figure 13:**
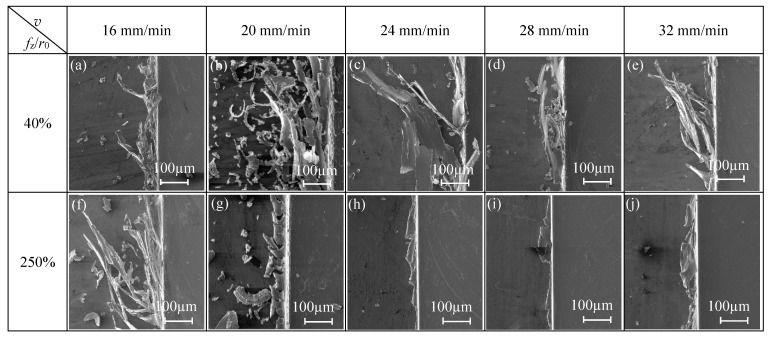
Changes of top burr morphology in the down milling side with respect to *v* in the size-effect zone (N17–N24).

When *f*_z_/*r*_0_ = 40%, cutting speed has an obvious effect on the top burr width in the down milling side. As shown in Figure 12a, the top burr width in the up milling side increases first and then decreases gradually when *v* increases from 16 mm/min to 32 mm/min, and the top burr width in the down millling side is larger than that in the up milling side. As shown in Figure 13a–e, the top burr morphology in the down milling side is mainly shown as a long strip block shape, and the top burr morphology in the up milling side mainly presents as a tearing block shape.

**Figure 14:**
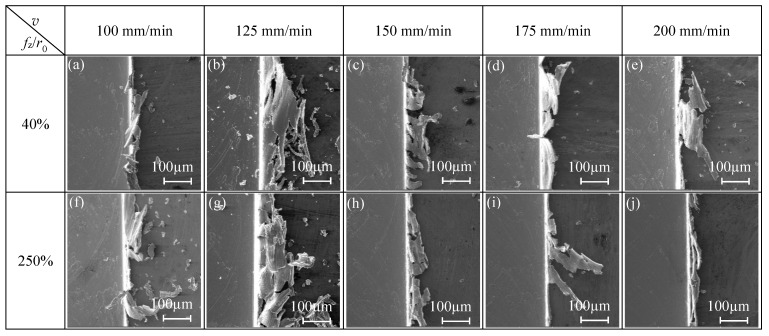
Changes of top burr morphology in the up milling side with respect to *v* in the size-effect zone (N17–N24).

When *f*_z_/*r*_0_ = 250%, Figure 12b shows that cutting speed has little effect on the top burr width in the micro-milling slot; the change of the top burr width in the up milling side is less than 30 μm when *v* increases from 100 mm/min to 200 mm/min. However, the top burr width in the down milling side is less than that in the up milling side when *v* increases from 12 mm/min. The top burr morphology in the down milling side changes from a long and thin strip burr to a small serrated burr (Figure 13f–j), and the top burr morphology in the up milling side changes from a large serrated burr to a small wavy burr (Figure 14f–j).

### 3.3. Optimization of Micro-Milling Parameters Based on Surface Roughness

**Figure 15:**
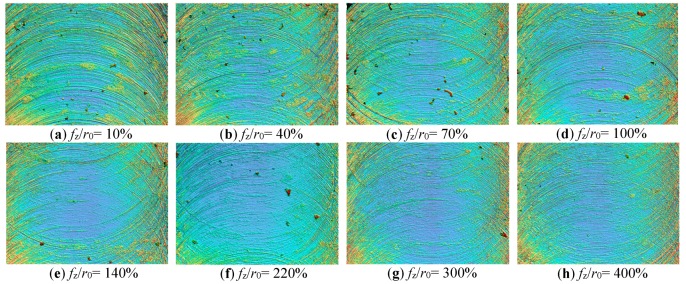
Surface morphology of the micro-slot bottom under different *f*_z_*/r*_0_ values measured by white light interferometer (Bruker contour GT-X): (**a**) *f*_z_/*r*_0_ = 10%; (**b**) *f*_z_/*r*_0_ = 40%; (**c**) *f*_z_/*r*_0_ = 70%; (**d**) *f*_z_/*r*_0_ = 100%; (**e**) *f*_z_/*r*_0_ = 140%; (**f**) *f*_z_/*r*_0_ = 220%; (**g**) *f*_z_/*r*_0_ = 300%; (**h**) *f*_z_/*r*_0_ = 400%.

There is an ideal *f*_z_/*r*_0_ cutting parameter range to make the surface roughness of the micro-slot bottom the micro-milling minimum. As shown in Figure 7b, when *f*_z_/*r*_0_ is less than 70%, the surface quality of the micro-slot bottom is poor, and the surface roughness of the micro-slot bottom increases rapidly with the decrease of *f*_z_/*r*_0_. This is because the micro-milling is in a strong size-effect zone, when *f*_z_ is less than *h*_min_, the material being machined mainly has elastic recovery, and the smaller the value of *f*_z_/*r*_0_, the stronger the squeezing and friction between the rake face of the micro-milling tool and the machined surface of the workpiece, thereby the changes in ploughing force are more significant. In addition, it is difficult to generate chips during the ploughing, and the workpiece material is slowly squeezed out after feeding multiple teeth, thus increasing the surface roughness, as shown in Figure 15a–c.

**Figure 16:**
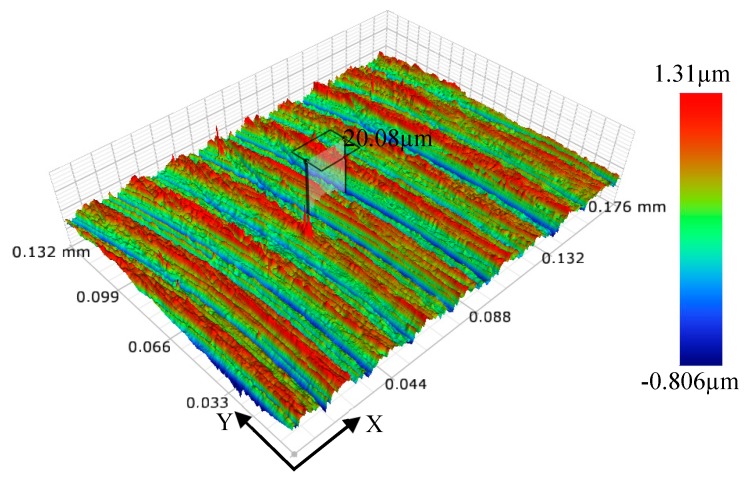
Three-dimensional topography of the micro-slot bottom surface measured by white light interferometer (Bruker contour GT-X).

When *f*_z_/*r*_0_ is larger than 220%, micro-milling is in a weak size-effect zone (as shown in Figure 7b). Shear force dominates the whole micro-milling process; the edge-size-effect of the tool is weakened; and surface roughness increases with the increase of *f*_z_/*r*_0_ value, but basically remains stable. As shown in Figure 16, when *f*_z_/*r*_0_ is 400%, *f*_z_ is far greater than *r*_0_ and *h*_min_; obvious regular tool marks were found on the machined surface. The distance between adjacent tool marks in the feed direction is 20.08 μm, which is close to the feed value (20 μm) of two neighboring teeth in a tool rotation circle. Consistent with the macro milling mechanism, this means there is an imprint of micro profile copying of micro-milling tool, resulting in a large *R*_a_ of the micro-slot bottom.

When *f*_z_/*r*_0_ is larger than 70% and less than 220%, micro-milling is in the transition zone of the edge-size-effect (Figure 7b), which means that micro-milling is in a process of ploughing transfer to shear. At this time, *f*_z_ is close to *h*_min_ which can just generate chips, with less adhesion of the chips on tools, thereby the machined surface is smooth with few defects and lower surface roughness at the slot bottom, as shown in Figure 15d–f. Therefore, the cutting parameters should be selected in the optimal roughness zone where *f*_z_/*r*_0_ is within 70%–220%, where the surface roughness can come down to 0.331 μm–0.44 μm, and an optimal *f*_z_/*r*_0_ value exists to minimize the surface roughness of the micro-slot bottom. The best *f*_z_/*r*_0_ value obtained from the N1–N8 group test in Table 1 is 140%, the surface topography is shown in Figure 15e.

*(1) Influence of Axial Cutting Depth on the Roughness of Micro-Slot Bottom*

**Figure 17:**
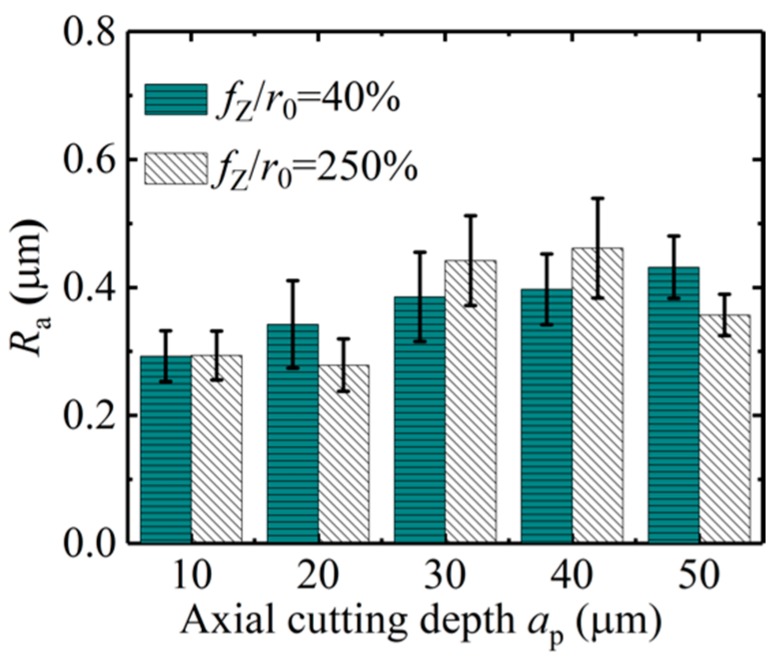
Surface roughness of the micro-slot bottom with respect to *a*_p_ in the size-effect zone (N9–N16).

**Figure 18:**
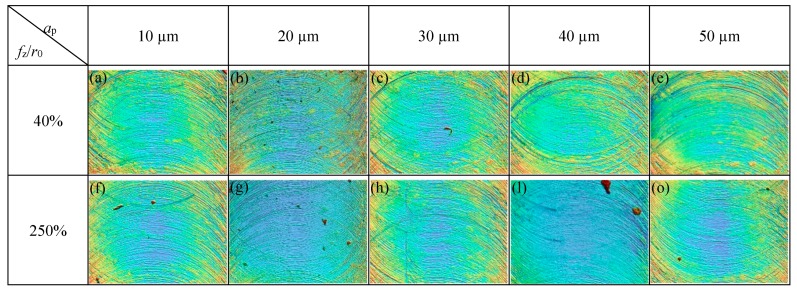
Surface morphology of the micro-slot bottom under different *a*_p_ (Bruker Contour GT-X).

The influence of the axial cutting depth on the surface roughness of the micro-slot bottom is shown in Figure 17; the cutting parameters used in the micro-milling test are listed in N9–N16 in Table 1. When *f*_z_*/r*_0_ is 40% and the axial cutting depth increases from 10 μm to 50 μm, the surface roughness of the micro-slot bottom gradually increases. This is because the micro-milling is in a strong size-effect zone and the edge-size-effect is significant, and notably squeeze and scratch exists between the tool and workpiece material, which causes the flank wear of the micro tool. Moreover, the increase of the axial cutting depth leads to the increase of the contact squeeze area, the volume of the squeezed workpiece material increases correspondingly, and the ploughing resistance increases. Although it is hard to form chips, many tool marks are formed on the surface of the micro-slot bottom, as shown in Figure 18a–e.

When *f*_z_*/r*_0_ is 250%, micro-milling is in the weak size zone. Figure 17 shows that, when the axial cutting depth increases from 10 μm to 40 μm, the *R*_a_ value of the micro-slot bottom increases from 0.28 μm to 0.42 μm correspondingly. This indicates that, although the tool is in contact with the workpiece material to generate stable chips under the main shear force, the resistance to plastic deformation of the workpiece material and the friction between the chips and the tool increase, and a small amount of chips left at the bottom of the micro-slot (Figure 18f–l), thus some of the chips are extruded twice by the tool flank (Figure 18)). When the axial cutting depth increases to 50 μm, the surface roughness decreases slightly. It is likely that the cutting edge of the milling tool is worn after the previous groups of experiments, which causes the larger cutting edge arc radius of the milling tool and, correspondingly, the *f*_z_*/r*_0_ value is reduced; the chip is easy to generate and to fall off at the edge of the micro-slot; and the regular and consistent tool imprints are formed at the bottom of the micro-slot, as shown in Figure 18o.

*(2) Influence of Cutting Speed on the Surface Roughness*

**Figure 19:**
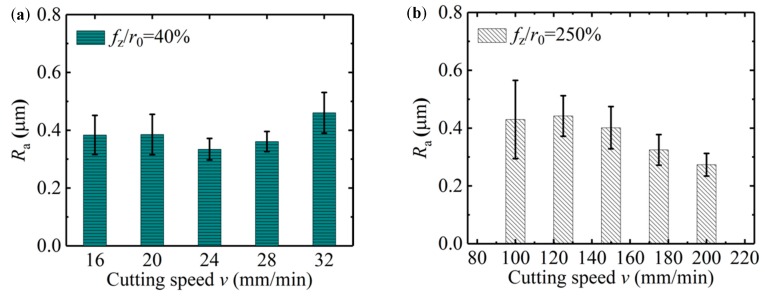
Surface roughness of the micro-slot bottom with respect to cutting speed *v* in the size-effect band (N17–N24): (**a**) *f*_z_/*r*_0_ = 40%; (**b**) *f*_z_/*r*_0_ = 250%.

The effect of cutting speed on the surface roughness is shown in Figure 19a; the cutting parameters of the micro-milling test are shown in Table 1 as N17–N24. When *f*_z_*/r*_0_ is 40%, *v* increases gradually from 16 mm/min to 32 mm/min, and the surface roughness value of the micro-slot bottom is small and slightly decreased. This shows that the micro-milling process is in a strong size-effect zone and no chips are formed, but with the increase of *v*, the contact time between the micro-milling tool and the workpiece material becomes shorter, and the material elastic deformation between the tool flank face and machined surface is small, the squeeze and friction effects reduce, and the plough cutting force has little deviations. When *v* increases to 200 mm/min, the tool may appear obviously runout, causing the sudden increase in the surface roughness value of the micro-slot bottom.

When *f*_z_*/r*_0_ is 250%, the surface roughness value of the micro-slot bottom gradually decreases with the increase of *v*, as shown in Figure 19b. This is because micro-milling is in the weak-edge-effect zone, the workpiece material forms stable chips under the effects of shear force, and regular tool marks are formed on the micro-slot bottom surface. In addition, the contact time between the tool and the chip becomes shorter when *v* is increased, the chips adhere less to the tool and separate more easily from the micro-slot bottom, leading to less residual chip on the micro-slot bottom.

## 4. Conclusions

This paper designed a single-factor micro-slot milling test for Al7075-T6 based on the quantitative analysis benchmark of *f*_z_/*r*_0_. Considering the influence of the edge-size-effect on the micro-milling process, based on the change of top burr width and surface roughness of the slot bottom with *f*_z_/*r*_0_, the strong size-effect zone, transition size-effect zone, and optimal size-effect zone are proposed, and the size-effect band is established. By adjusting *f*_z_/*r*_0_ to make micro-milling break away from the strong edge-size-effect zone, the cutting parameters are thereby optimized, and reduction of the surface roughness of the micro-slot bottom and reduction of the burrs can be achieved. The following conclusions can be drawn.

**(1)***f*_z_/*r*_0_ provides an analytical basis for quantitative study on the influence of cutting parameters on burr formation and surface quality. When the *f*_z_/*r*_0_ ratio gradually increases, the top burr width increases first and then decreases; however, the surface roughness of the micro-slot bottom decreases first and then increases. The optimal cutting parameters should be selected in the size-effect band that is least affected by the edge-size-effect in micro-milling. When *f*_z_/*r*_0_ is 400%, the number of burrs is the least and the top burr width is the smallest; when *f*_z_/*r*_0_ is 140%, the surface roughness of the micro-slot bottom is the smallest.

**(2)** When *f*_z_/*r*_0_ is less than 40%, micro-milling is in the strong size-effect zone, and machined surface material with elastic deformation contacts the flank face of the micro tool to form a serious squeezing and ploughing, resulting in a large and non-linear milling force; the surface quality is poor, the top burr width changes significantly, and the top burr width in the down milling side is much larger than that in the up milling side.

When *f*_z_/*r*_0_ is in the range of 70%–220%, micro-milling is in the transition size-effect zone, and the contact between the workpiece material and the micro-milling tool results in elastic deformation and shear deformation at the same time, thus forming the shear resistance and friction resistance. The surface quality is the best at this situation, although the chip formation is not stable, and plentiful burrs are generated on the top of the micro-slot; the top burr width in the down milling side is much larger than that in the up milling side.

When *f*_z_/*r*_0_ is greater than 220%, micro-milling is in the weak size-effect zone, the deformed material contacts with the flank face of the micro-milling tool to form stable chips, the number of burrs is significantly reduced, and the top burr width is reduced on the top of the micro-slot; the top burr width in the down milling side is gradually smaller than that in the up milling side. The tool imprint phenomenon appeared on the milled surface, the surface quality was the worst, and the surface roughness of the micro-slot bottom gradually increased.

**(3)** Feed rate per tooth (*f*_z_) have a significant effect on the top burr width and surface roughness of the micro-slot bottom, while the axial cutting depth (*a*_p_) and cutting speed (*v*) have a small effect on the top burr width and surface roughness of the micro-slot bottom. Increasing *f*_z_ or *a*_p_ can reduce the top burr width, and the micro-milling gradually breaks away from the edge-size-effect zone. Micro-milling is in the transition size-effect zone and *R*_a_ of the micro-slot bottom is good, and increasing *f*_z_ or increasing *a*_p_ can reduce *R*_a_ of the micro-slot bottom.

**(4)** There are four kinds of top burr shapes during the micro-milling process: continuous serration, continuous long whisker, curl wave, and tear flag. The number of chips in the up milling side is small and most of them form curly top burrs, and the number of chips on the down milling side is large and most of them form long whisker or serration top burrs.

## Figures and Tables

**Table 1:** Cutting parameters used in the experiment.

Test	Cutting Speed *v* (mm/min)	Feed Rate Per Tooth *f*_z_ (µm/tooth)	Axial Cutting Depth *a*_p_ (µm)	Tool Extend Length *l* (mm)
N1	5	0.25	30	20
N2	20	1	30	20
N3	30	1.5	30	20
N4	50	2.5	30	20
N5	70	3.5	30	20
N6	110	5.5	30	20
N7	150	7.5	30	20
N8	200	10	30	20
N9	20	1	10	20
N10	20	1	20	20
N11	20	1	40	20
N12	20	1	50	20
N13	125	6.25	10	20
N14	125	6.25	20	20
N15	125	6.25	40	20
N16	125	6.25	50	20
N17	16	1	30	20
N18	24	1	30	20
N19	28	1	30	20
N20	32	1	30	20
N21	100	6.25	30	20
N22	150	6.25	30	20
N23	175	6.25	30	20
N24	200	6.25	30	20
